# Unraveling the microbial diversity of bovine liver abscesses: isolation, identification, and genomic characterization of the *Bacteroides* found in hepatic lesions

**DOI:** 10.1128/spectrum.00423-25

**Published:** 2025-04-17

**Authors:** R. J. Gruninger, M. L. McCormack, N. C. Chomistek, R. Zaheer, T. A. McAllister

**Affiliations:** 1 Lethbridge Research and Development Centre, Agriculture and Agri-Food Canada, Lethbridge, Alberta, Canada; Michigan State University, East Lansing, Michigan, USA

**Keywords:** abscesses, *Bacteroides*, bovine, liver, intra-abdominal infection, ruminant, genomics, metagenomics

## Abstract

Liver abscesses in cattle reduce animal performance, increase the environmental footprint of beef production, and cause significant economic losses. The low pH of the rumen resulting from the consumption of high grain diets damages the rumen epithelium and facilitates the translocation of opportunistic pathogens from the gastrointestinal tract into the bloodstream where they can colonize the liver, causing infection. Recently, 16s rRNA sequencing has revealed that 25%–50% of liver abscess microbiomes have prominent levels of *Bacteroides*. Due to the inability to reliably classify amplicon sequences beyond the genus level, the identity of these microbes remains unknown. We have employed a combination of culture-independent and culture-based methods to isolate and identify the *Bacteroides* associated with liver abscesses in cattle. Shotgun metagenomic sequencing and assembly of metagenome-assembled genomes generated four high-quality genomes, two of which were putatively identified as *Bacteroides*. These microbes were subsequently isolated from the purulent material of liver abscesses. Whole-genome sequencing conclusively identified these isolates as *Bacteroides pyogenes* and a previously unknown species of *Bacteroides,* revealing distinct differences from *Bacteroides* typically found in the gut. Carbohydrate utilization assays revealed that both organisms metabolize glycogen and glycosaminoglycans found in the extracellular matrix of the liver but display differences in substrate specificity. These data not only identify *Bacteroides* found in bovine liver abscesses but also provide new insights into the potential role that these organisms may play in this production-limiting disease.

**IMPORTANCE:**

Liver abscesses (LAs) are commonly found in cattle raised in feedlots and result from a bacterial infection of the liver. Not only are LAs a concern for animal health, but they also impact growth efficiency, animal welfare, and cost the North American beef industry upwards of $120 million per annum. Recently, it has been found that 25%–50% of liver abscess microbiomes have prominent levels of *Bacteroides*; however, to date, the biological relevance in LA pathogenesis and the identity of these bacteria are unknown. This research describes the isolation, identification, and genomic characterization of the *Bacteroides* found in bovine liver abscesses. These data provide a critical foundation for expanding our knowledge of the potential role *Bacteroides* play in liver abscess development and could contribute to the identification of novel targets for developing treatments to prevent this important production-limiting disease.

## INTRODUCTION

Liver abscesses (LAs) are commonly found in cattle raised in feedlots and result from a bacterial infection of the liver. Despite the inclusion of antimicrobials in the diets of feedlot cattle, the prevalence of liver abscesses in Canada is increasing and has risen from 9.9% in 2010–2011 to 19.3% in 2016–2017 (1). In extreme circumstances, over 90% of cattle in some cohorts have been found to have LA (2). LAs represent a significant economic burden for beef producers, with an estimated cost of >$68 million per annum in Canada and upwards of $400 million per annum in the United States (1). Severe LAs are also known to reduce animal performance, resulting in a 5%–15% reduction in average daily gain and a 9.7% reduction in feed efficiency (3–5). Based on the significant impact that LAs have on animal performance, the true cost of LA to producers and consumers is likely much greater than current estimates.

It is hypothesized that LAs develop due to rapid microbial fermentation of starch in the rumen and/or hindgut, which can lead to clinical and/or sub-clinical acidosis. The production of volatile fatty acids at a rate faster than they can be absorbed and metabolized lowers rumen pH and disrupts the integrity of rumen and intestinal epithelium. These areas of damage enable opportunistic pathogens, like *Fusobacterium necrophorum,* to translocate to the liver via the portal system (6). The gram-negative bacteria *F. necrophorum* have long been regarded as the primary etiological agent of LA, being almost universally found among culture-based LA microbiota studies (2, 6, 7). Recent metataxonomic surveys suggest that LA microbial communities are more complex than initially thought, with multiple bacteria likely playing a role in LA etiology (7–10). The recent discovery of *Fusobacterium*- or *Bacteroides*-dominated LA microbiome sub-types has challenged the long-held view of *F. necrophorum* as the primary etiological agent of LA (6, 7). While the biological significance of the *Bacteroides* commonly found in LAs is unclear, identification, isolation, and genomic characterization of these microbes can provide a foundation for determining what role, if any, they may play. To date, the species-level identification of the *Bacteroides* found in LA is currently unknown because amplicon sequencing cannot accurately identify bacteria beyond the genus level.

*Bacteroides* are commensal, obligate anaerobes with an essential role in gut health and digestion, and they are among the most abundant taxa within the gut microbiome of both monogastric and ruminant livestock (11–13). *Bacteroides* are capable of utilizing a range of host and non-host glycans as substrates, playing a key role in carbohydrate metabolism (14). By encoding a diverse array of polysaccharide catabolic genes and regulating the transcription of gene clusters known as polysaccharide utilization loci (PUL), *Bacteroides* can shift glycan utilization *in vivo* based on nutrient availability (15, 16). Examining the presence and/or absence of carbohydrate active enzymes (CAZymes) and PULs can provide clues about the specific role that *Bacteroides* play within an environment (15, 16).

Despite their beneficial role in the gut, *Bacteroides* can cause severe infections when they breach the GI tract, leading to bacteremia and the formation of abscesses in a variety of tissues, including the abdomen, brain, liver, pelvis, and lungs (17). While *Bacteroides fragilis* is the most commonly isolated species from clinical specimens, other species, including *Bacteroides vulgatus*, *Bacteroides uniformis*, *Bacteroides thetaiotaomicron*, *Bacteroides ovatus*, *Bacteroides stercoris*, *Bacteroides caccae, Bacteroides pyogenes,* and *Bacteroides heparinolyticus,* have been isolated from infections (17–20). Pathogenic *Bacteroides* encode virulence factors (VFs), including extracellular enzymes, hemolysins, capsular polysaccharides, antibiotic resistance, and adhesins (21). The secretion of hydrolytic enzymes, including proteases, hyaluronidase, chondroitin sulfatase (CS), elastase, fibrinolysin, and lipases, is thought to contribute to the destruction of the extracellular matrix (ECM) (17, 22). *Bacteroides* in the gut are commonly exposed to antimicrobials, leading to the acquisition of a diverse range of antibiotic resistance mechanisms, with multidrug-resistant clinical isolates being more frequently isolated (21, 23, 24). The capsule polysaccharides of *B. fragilis* are another VF and have been found to initiate an immune response in the host that leads to the formation of an abscess at the site of infection (17, 21, 25). The fibrous membrane around the infection site is a host defense mechanism to isolate and contain invasive bacteria. It is unclear if all *Bacteroides* species elicit this immune response, but *B. thetaiotaomicron* capsular polysaccharides have also been found to modulate the host immune response, interfering with bacterial clearance by T-cells (26).

The potential for commensal *Bacteroides* to cause serious infections in a variety of tissues, and the recent recognition that members of this genus may play an important role in LA pathology, make the identification and characterization of the *Bacteroides* found in LA a significant knowledge gap. Herein, we describe culture-independent and culture-based studies to isolate and identify *Bacteroides* from bovine LA.

## MATERIALS AND METHODS

### Collection of liver abscess samples for *Bacteroides* metagenomic and culturing experiments

Liver abscess samples used for the shotgun metagenomic experiments were obtained from a commercial feedlot experiment examining the impact of altering the timing of tylosin treatment (27). Animals in this experiment were fed a finishing diet consisting of 85.8% concentrate, 11.5% roughage, and 2.8% supplement, with the concentrate portion consisting of 70% corn and 15.8% tempered rolled barley or wheat. Full details of animal management can be found in Davedow et al. (27). After slaughter at a commercial abattoir, livers were evaluated by a certified CFIA inspector using the Elanco Liver Scoring System (0, A, and A+) and transported to a mobile laboratory facility that was positioned near the abattoir’s slaughter operations wing exit. Liver samples were collected aseptically from a subset of animals for metagenomics and bacterial isolation studies. The liver surface was first disinfected with 70% ethanol, and a sterile scalpel was used to excise abscesses. For metagenomics, up to 3 cm^3^ of abscess tissue was placed in a 50 mL Falcon tube and immediately flash-frozen in liquid nitrogen and stored at −80°C. Liver abscess samples were collected from another feedlot finishing study that examined the effects of protein level and processing methods of wheat (28). The liver samples that were used for the isolation of *Bacteroides* employed the same methods, but tissue and/or purulent material was not frozen, and cultures were inoculated within 4 hours of sample dissection. Animals in this experiment were fed a finishing diet consisting of 85% wheat, 10% barley silage, and 5% supplement (DM basis). Monensin was included in all diets at a concentration of 44 mg/kg of body weight, with no antimicrobials used to control liver abscesses.

### Extraction and purification of DNA from liver abscesses

A random subset of 16 tissue samples collected by Davedow et al. (27) was used to extract liver abscess metagenomic DNA following selective depletion of bovine DNA using the methods described by Charalampous and colleagues (29). Three hundred twenty-five milligrams of abscessed tissue was added to a sterile 2.0 mL safe-lock tube with 1 mL PBS, vortexed for 30 s, and centrifuged at 500 × *g* at 4°C for 5 min. The resulting supernatant was centrifuged again at 6,000 × *g* for 15 min to obtain a cell pellet. The pellet was washed with 250 µL PBS (pH 7.4), followed by a 10 min incubation of the pellet with 200 µL of 5% saponin (Sigma Aldrich, Oakville, ON, Canada) to lyse host cells. Following the 10 min incubation, 350 µL of sterile deionized water was added to the mixture and incubated at room temperature for 30 s, and osmotic shock was used to lyse host cells by adding 12 µL of 5 M NaCl. The mixture was then centrifuged at room temperature for 5 min at 6,000 × *g,* with the resulting pellet resuspended in 100 µL PBS buffer. Bovine DNA from lysed liver cells was digested for 15 min at 37°C by the addition of 250 Units HL-SAN DNase kit according to the manufacturer’s instructions (ArticZymes, Cedarlane, Burlington, ON, Canada). Host DNA-depleted cells were then pelleted by centrifugation at room temperature for 3 min at 6,000 × *g* and washed twice with 1 mL of PBS. The cell pellet was used to extract metagenomic DNA using the DNeasy Blood and Tissue Kit according to the manufacturer’s instructions (Qiagen Inc., Toronto, ON, Canada). DNA quality was assessed using a Quant-iT PicoGreen kit (Thermo Fisher Scientific, Mississauga, ON, Canada). The purity of the DNA was determined by measuring the ratios of absorbance at 260/280 and 260/230 using a NanoDrop spectrophotometer (Thermo Fisher Scientific, Waltham, MA, USA). Samples with a 260/280 ratio value between 1.7 and 2.0 and a 260/230 ratio between 2.0 and 2.2 were considered acceptable for downstream analysis. Purified DNA was stored at −80°C until sequenced.

### Metagenomic sequencing and bioinformatic analysis

Sequencing and library generation were completed at the McGill Genome Sequencing Centre (Montreal, QC, Canada). Shotgun libraries were generated using NEBNext Ultra II DNA Library Prep (New England Biolabs, Whitby, ON, Canada). The resulting libraries were sequenced using an Illumina NovaSeq6000 S4 platform to obtain an average of 72 million 150 base paired-end (PE) reads per sample. Sequence quality was assessed with FASTQC, and reads were processed with Trimmomatic version 0.39 (30) to remove adapter sequences and low-quality reads. The parameters ILLUMINACLIP:TruSeq3-SE.fa:2:30:10:3:TRUE LEADING:3 TRAILING:3 SLIDINGWINDOW:4:20 MINLEN:36 were used for QC and sequence trimming. The resulting reads were mapped to the *Bos tarus* ARS-UCD1.3 genome using Bowtie2 to identify any bovine DNA (31, 32). Unmapped reads were then mapped to the *Homo sapiens* genome GRCh38.p14 to remove any human DNA (33). The remaining unmapped reads were used as input to SqueezeMeta to generate metagenome-assembled genomes (MAGs) (34). Co-assembly mode was used in SqueezeMeta to generate contigs with the assembler Megahit using the parameters “-min-count 3 --k-min 21 --k-max 121 --k-step 10 --prune-level 3 -t 24 --min-contig-len 1000.” The resulting contigs were binned into genomes using Metabat2 (35), Maxbin2 (36), and Concoct (37) and dereplicated with DAStool (38). Individual samples were mapped to MAGs with Bowtie2 (32) using default settings. Prodigal was used to identify ORFs, and functional annotation was assessed using PFAM, KEGG, and GO databases (39). DESeq2 was used to identify differentially abundant KEGG functional categories between abscess subtypes. Bin quality was assessed with CheckM, and only bins classified as high quality (>90% complete with <5% contamination) were considered for further downstream analysis (40). MAG annotations were exported to R and visualized using SQMtools (41).

### Isolation of *Bacteroides*

Abscesses collected for bacterial isolation were transported to the lab on ice within 4 hours and cut open using a sterile scalpel. Purulent material from the center of the abscess was inoculated into Hungate tubes containing 10 mL anaerobic Columbia broth (BD Difco). The inoculated media were homogenized and diluted 1:10, 1:100, and 1:1,000, and a 100 µL aliquot was directly plated onto Columbia broth agar (BD Difco) supplemented with 5% sheep’s blood (CBS agar) in an anaerobic chamber. Plates were incubated for 96 hours at 37°C in an anaerobic chamber with 85% N_2_, 10% CO_2_, and 5% H_2_. Purulent material was also inoculated into a Hungate tube containing Columbia broth supplemented with 0.2 g/mL L-Cysteine, 1 mg/mL resazurin, 5 µg/mL vancomycin, and 5% sheep’s blood (CBS broth) and incubated for 72 hours at 37°C prior to plating to enrich for microbes in the purulent material. Enrichment samples were then serially diluted (1:10, 1:100, and 1:1,000), and 100 µL was plated onto CBS agar plates. Enrichment plates were incubated in an anaerobic chamber for 48 hours. Plates were manually inspected, and well-resolved colonies with distinct phenotypes (shape, size, and color) were inoculated into a 96-well plate containing CBS broth and incubated for 48 hours in an anaerobic chamber. Wells showing growth were sub-cultured onto 60 mm CBS agar plates and anaerobically incubated for 48 hours at 37°C.

Isolates were first screened using a *Fusobacterium-*specific primer set (Fuso1: 5′-GAG AGA GCT TTG CGT CC-3′, Fuso2: 5′-TGG GCG CTG AGG TTC GAC-3′, Wlf1: 5′-GCACCATTTTGAGCGCGT-3′, Wlf2: 5′-AGGTGCTTCTTCCACAGC-3′, lktA1: 5′-AATCGGAGTAGTAGGTTCTG-3′, and lktA2: 5′-CTTTGGTAACTGCCACTGC-3′) to rapidly identify *Fusobacterium* isolates (42, 43). Isolates that did not show amplification were then screened by Sanger sequencing of their partial 16S rRNA gene with the primers 27F (5′-AGAGTTTGATCMTGGCTCAG-3′) and 806R (5′-GGACTACHVGGGTWTCTAAT-3′) at the McGill Genome Sequencing Centre (44, 45). 16S rRNA sequences were searched against the NCBI database using blastn, and isolates putatively identified as *Bacteroides* were selected for whole-genome sequencing.

### Whole-genome sequencing of isolates

Isolates putatively identified as *Bacteroides* underwent a minimum of two rounds of re-isolation on CBS agar to ensure purity. Genomic DNA was extracted from a 5 mL overnight culture grown in CBS broth under anaerobic conditions. DNA was purified with Genomic-tip 20/G according to the manufacturer’s instructions (Qiagen, Toronto, ON, Canada). Short-read sequencing of a NexteraXT DNA library was performed on the Illumina MiSeq platform with a MiSeq reagent V3, 2 × 300 bp PE kit according to the manufacturer’s protocol (Illumina, San Diego, CA, USA). Quality control of Illumina reads was conducted with Trimmomatic version 0.39 (30). Nanopore sequencing libraries were prepared using the Ligation Sequencing Native Barcoding Kit 24 V14 without DNA fragmentation (NBD114.24, Oxford Nanopore, Oxford, UK). Long-read sequencing was performed with a minION using an R10.4.1 flowcell as directed by the manufacturer (Oxford Nanopore, Oxford, UK). MinKNOW Core 23.04.6 was used for flowcell signal processing and base calling. Adapter trimming and read QC were performed by Guppy 6.5.7, and reads passing the default quality cutoff were assembled *de novo* with Flye version 2.9.5 (46). Hybrid genome assemblies were generated with Unicycler version 0.5.1 using default settings (47), and genome quality was assessed using Quast version 5.2.0 (48).

### Functional annotation

Genomes were annotated using Prokka (version 1.14.6) (49). Proteins identified with Prokka were further annotated with InterProScan (version 5.59-91.0) (50) using Pfam (51), FunFam (52), TIGRFAM (53), HAMAP (54), and eggNOG5 (55). Metabolic pathway presence and completeness were predicted by Distilled and Refined Annotation of Metabolism (DRAM) (56). Virulence factors were identified with the virulence factor database (VFDB) (accessed: 2 July 2024) (57). Sulfatases were identified and classified using the sulfatases sequences version 2.3.1 database using SulfAtlas (version 2.3.1) (58). Carbohydrate active enzymes were identified using dbCAN3 (59) and classified based on the CAZy database (accessed 1 November 2024) (60). PULs were identified by scanning the genome annotation files for the SusC/D PUL markers and other boundary outlining rules previously defined (61) and were visualized using the packages gggenes (version 0.5.1) (62) and ggplot2 (version 3.5.1) (63). Enzymatic activity of CAZymes was predicted with SACCHARIS 2.0 (64). Secreted proteins were predicted with SignalP 6.0 (65), and subcellular localization was predicted with DeepLocPro 1.0 (66).

### Phylogenetic analysis **of** *Bacteroides* LA isolates

Taxonomy of MAGs and isolate genomes was determined using two independent tools: Type Strain Genome Server ([TYGS] dsmz.de, accessed 25 April 2024 [67]; and GTDB-tk version 2.4.0 [68, 69]). Query sequences were aligned to the GTDB (version r214) bac120 (69) database, and evolutionary distance branch lengths were used to create the *Bacteroides* phylogenetic tree. Phylogenetic trees were rooted with *Prevotella melaningenica* (GCF_000144405.1) as an outgroup in R (version 4.2.1) using R studio (2022.12.0) with the “root” function of the ape package (version 5.7–1) (70) and were visualized using iTOL (version 6.9.1) (71). Average nucleotide identity (ANI) was conducted using Fast whole-genome similarity (ANI) estimation (FastANI) (version 1.34) to compare genome sequences to the closest *Bacteroides* type strain(s). An ANI score of ≥95% was used to demarcate species (72). FastANI results were visualized using the pheatmap package (version 1.0.12) (73).

### Growth of *Bacteroides* LA isolates on α-glucan and GAG substrates

*B. purulensis* and *B. pyogenes* were grown in customized chopped meat medium (CM) (74) in an anaerobic chamber for 16 hours, after which the cells were pelleted by centrifugation (4,000 × *g* for 10 min) and washed twice with a carbohydrate-free version of CM (MM) before being diluted to an OD_600nm_ of 0.1. A 1% (wt/vol) carbohydrate solution consisting of glucose (G7021; Sigma, USA), glycogen (G0885; Sigma, USA), starch (S9765; Sigma, USA), glucuronic acid (G5269; Sigma, USA), N-acetylglucosamine (A8625; Sigma, USA), N-acetylgalactosamine (A2795; Sigma, USA), hyaluronic acid (HA) (FH145201; Biosynth, USA), chondroitin sulfate (YC04273; Biosynth, USA), and heparin (H5515; Sigma, USA) was used for carbohydrate growth assays. Wells of a 96-well microtiter plate (353072; Corning, USA) were filled with 100 µL of sterilized carbohydrate solution and 100 µL of bacterial inoculant (OD_600nm_ of 0.1). Negative control wells consisted of 100 µL of 2× MM combined with 100 µL of 1% carbohydrate and were used to normalize growth curves. Additional media controls were conducted, where the growth of each LA isolate was assessed in rich media (CM) and the respective carbohydrate-free MM. Polyurethane Breath-Easy sealing membranes (Sigma, USA) were used to seal the plates. Absorbance (OD_600nm_) of each well was measured with an Alto plate reader (Cerillo, USA) and was recorded on a microSD card every 10 min for 72 hours. The mean (± standard deviation) for each condition (*n* = 4) was plotted in Excel.

## RESULTS

### Metagenomic sequencing and MAG assembly

Metagenomic DNA extraction and sequencing were conducted on 16 randomly selected liver abscess samples collected from commercial feedlot cattle (27). The results of sequencing and read quality control were mapped against the bovine and human genomes. A total of 214.2 Gb of sequence data did not map to the bovine or human genomes, with a range of 7.3–21.6 Gb of sequence data that are putatively microbial in origin. Two samples contained >99% eukaryotic DNA, indicating that host DNA depletion was ineffective for these samples; as a result, they were not considered further. Co-assembly of the remaining 14 samples generated 4,994 contigs >1,000 bp (19.7 Gb) with an *N*_50_ of 9,190 bp. The longest contig in the assembly was 284 kb. Of the 11 MAG bins, only four were high quality based on the generally accepted classification standards for MAG completeness and contamination (Table 1) (75).

**TABLE 1:** Taxonomic summary, genome size, completion, and contamination statistics of high-quality MAGs generated from abscessed liver tissue

Bin	Taxonomy	Size (bp)	Contigs	Completeness (%)	Contamination (%)
bin 1	p_*Bacteroidota* c_*Bacteroidia* o_*Bacteroidales* f_*Bacteroidaceae* g_*Bacteroides* s_*pyogenes*	2,886,811	67	91.4	0.7
bin 2	p_*Bacteroidota* c_*Bacteroidia* o_*Bacteroidales* f_*Bacteroidaceae* g_*Bacteroides*	2,631,979	269	93.6	1.0
bin 3	p_*Fusobacteriota* c_*Fusobacteriia* o_*Fusobacteriales* f_*Fusobacteriaceae* g_*Fusobacterium* s_*necrophorum*	2,338,561	452	92.4	3.5
bin 4	p_*Actinomycetota* c_*Coriobacteriia* o_*Coriobacteriales* f_*Atopobiaceae* g_*Atopobium*	1,610,340	31	100	0

### Distribution of MAGs among LA samples

**Fig 1:**
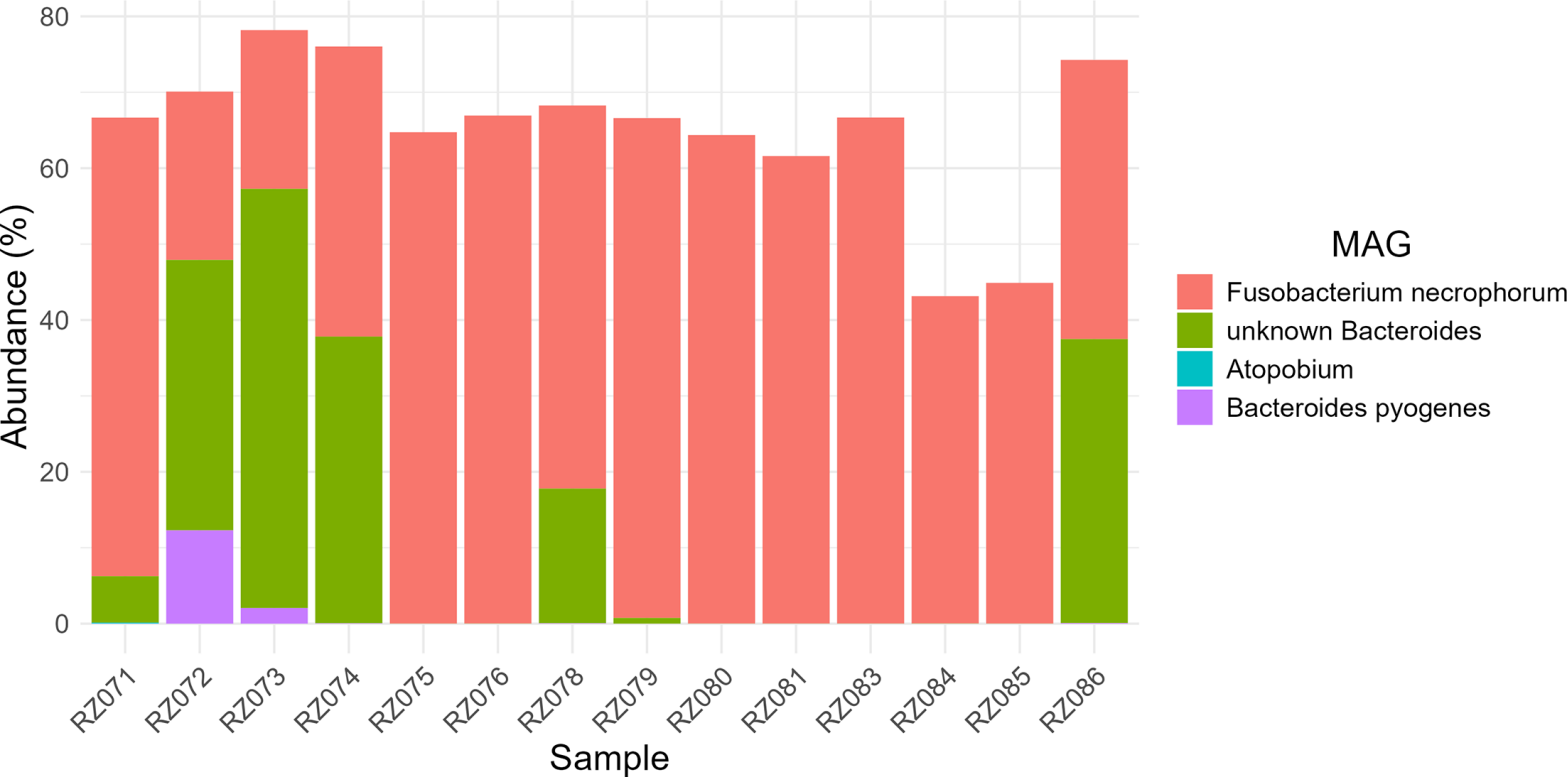
Abundance profile of MAGs in abscessed liver tissue samples.

The relative abundance of *Bacteroides pyogenes* (bin 1) and an unknown species of *Bacteroides* (bin 2) ranged from 17.8% to 57.3% in five of the abscess samples (Fig. 1). The other nine samples were dominated by *Fusobacterium necrophorum* (bin 3). The *Fusobacterium* MAG had a high level of heterogeneity consistent with the presence of more than one subspecies of *F. necrophorum* that cannot be distinguished using this approach. A second *Fusobacterium* bin was assembled; however, it was only 23.5% complete, with 3.3%–11% of the reads mapping to it and, therefore, was excluded from downstream analysis. A MAG from the genus *Atopobium* within the phylum *Actinomycetota* was also observed in a single sample (RZ071) at a relative abundance of <0.1% and was not analyzed further. An average of 17.4% (range 4.1%–23.8%) of the contigs in the samples could not be classified.

### Differentially abundant functional categories in *Fusobacterium* and *Bacteroides*: *Fusobacterium*-dominated abscesses

In agreement with recent metataxonomic studies of LAs, the abscesses examined were categorized as: (i) *F. necrophorum* subtype (F-type), and (ii) *Bacteroides* and *Fusobacterium* subtype (BF-type, RZ071, RZ072, RZ073, RZ074, RZ078, and RZ086 in Fig. 1). Twenty-one KEGG functions were found to be more abundant in F-type abscesses, including iron transport systems, a multi-sugar ABC transporter, filamentous hemagglutinin, and several bacterial stress response proteins (Data S1). Six hundred forty-seven KEGG functions were more abundant in BF type abscesses, including several proteases, heparin lyase, hyaluronoglucosaminidase, sulfatases, glycogen phosphorylase/synthase, Fe-transporter, a macrolide efflux protein, TetR transcription regulator, carbohydrate transporters, carbohydrate active enzymes, and hemolysin/hemolysin transcription regulators. Several of the functions that were most differentially abundant in BF-type abscesses are involved in carbohydrate metabolism and degradation of glycosaminoglycans (GAGs) found in the extracellular matrix, including heparan-sulfate lyase (EC:4.2.2.8), hyaluronoglucosaminidase (EC:3.2.1.35), chondroitin-sulfate-ABC endolyase/exolyase (EC:4.2.2.20 4.2.2.21), unsaturated chondroitin disaccharide hydrolase (EC:3.2.1.180), and sulfatase (EC:3.1.6.-).

### Isolation of *Bacteroides* from liver abscess

Direct plating and enrichment culture techniques were carried out on purulent material from six liver abscess samples. A total of 260 colonies were selected and sub-cultured. Of the isolates, 53% were putatively identified as *Fusobacterium* sp. using a PCR-based screening method with *Fusobacterium*-specific primers. Twelve non-*Fusobacterium* isolates, obtained from two samples, were identified as *Bacteroides* by a blastn search of a partial 16S rRNA sequence. The top blastn hit for six isolates from one sample was *B. pyogenes* JCM 6294 (GenBank accession AB200229), and for six isolates from a second sample, it was *Bacteroides heparinolyticus* ATCC 35895 (GenBank accession L16487.1).

### Whole-genome sequencing and phylogenetic analysis of *Bacteroides* isolates

**Fig 2:**
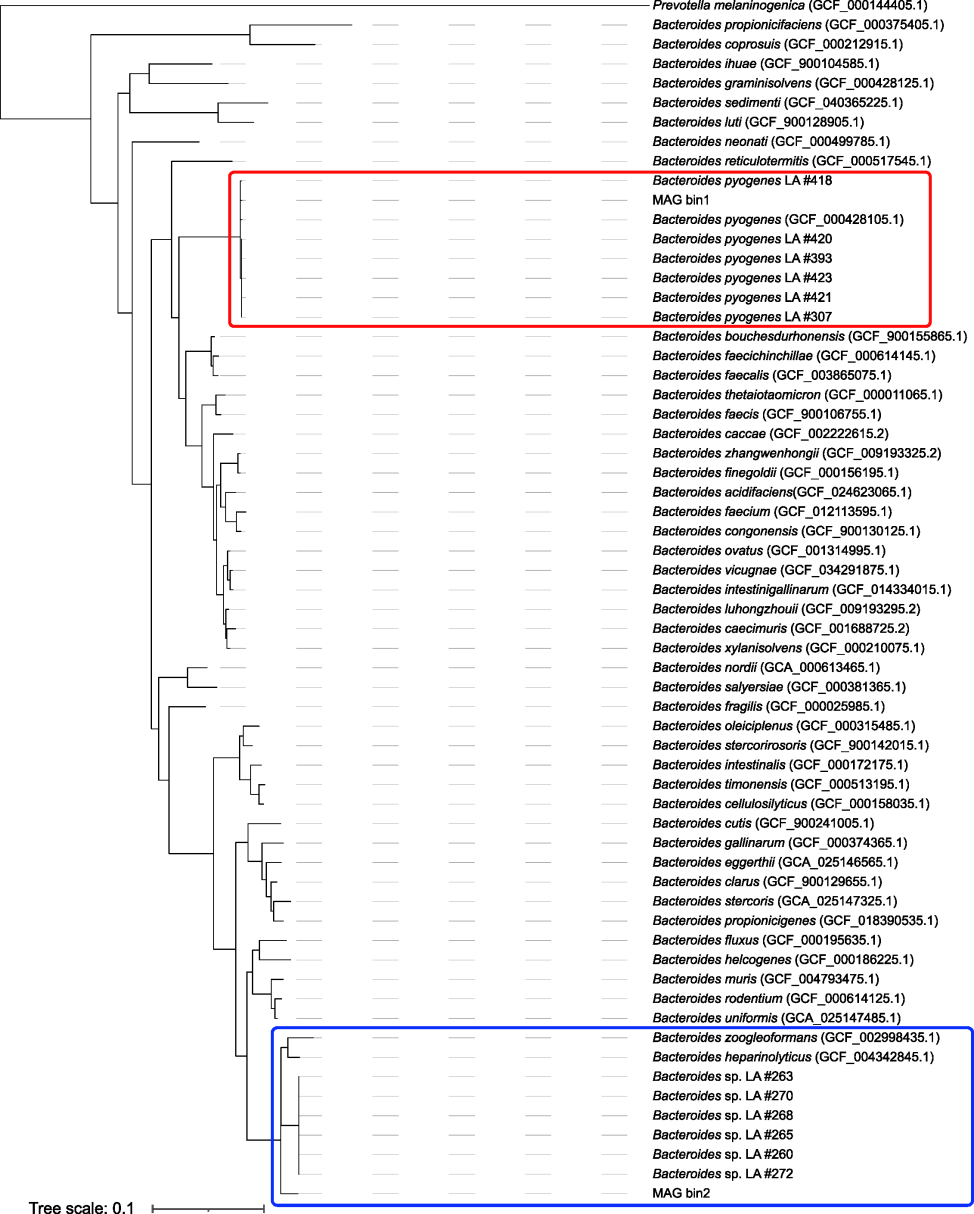
Whole genome phylogenetic tree of *Bacteroides* isolates and MAGs from liver abscesses. The clade containing *B. pyogenes* LA isolates (#307, 393, 418, 420, 421, and 423) and MAG bin1 is highlighted with a red box. The clade containing LA isolates from an unknown *Bacteroides* species (#260, 263, 265, 268, 270, and 272) and MAG bin2 is highlighted with a blue box. Only genomes from classified species of *Bacteroides* and *Prevotella melaninogenica* (outgroup) were included in the tree.

To conclusively identify the *Bacteroides* isolates, whole-genome sequencing was performed (Table 2). Whole genome-based phylogenetic analysis with GTDB-Tk revealed that the isolates form two clusters in the phylogenetic tree (Fig. 2). Isolates #307, 393, 418, 420, 421, 423, and MAG bin1 formed a clade and clustered with *B. pyogenes* DSM 20611 type strain (GCF_000428105.1) (Fig. 2, red box). Whole genome average nucleotide identity between *B. pyogenes* type strain, isolates #307–423, and MAG bin1 was >98% (Fig. S1A). There was >98% sequence similarity between the *B. pyogenes* isolates and MAG bin1 and >99% sequence similarity among the six *B. pyogenes* isolates.

**TABLE 2:** Genome assembly statistics and genomic features of *Bacteroides* isolated from liver abscesses^*a*^

Bacterial strain	Total genome length (bp)	No. of contigs	GC content (%)	*N*_50_ (bp)	No. of identified protein features
*Bacteroides pyogenes* #307*	3,521,837	1	45.92	3,521,837	2,966
*Bacteroides pyogenes* #393*	3,551,752	3	45.9	3,521,799	2,997
*Bacteroides pyogenes* #418*	3,520,369	1	45.92	3,520,369	2,989
*Bacteroides pyogenes* #420	3,521,788	1	45.92	3,521,788	2,985
*Bacteroides pyogenes* #421*	3,521,572	1	45.92	3,521,572	2,969
*Bacteroides pyogenes* #423*	3,521,845	1	45.92	3,521,845	2,972
*Bacteroides* sp. #260	3,032,037	2	47.42	3,030,303	2,553
*Bacteroides* sp. #263	3,031,524	1	47.41	3,031,524	2,530
*Bacteroides* sp. #26*5*	3,030,331	1	47.42	3,030,331	2,526
*Bacteroides* sp. #268	3,029,060	1	47.42	3,029,060	2,529
*Bacteroides* sp. #270	3,029,024	1	47.42	3,029,024	2,536
*Bacteroides* sp. #272*	3,052,543	2	47.43	3,029,036	2,556

^*a*^ * denotes that bold bridging setting was used for unicycler co-assembly.

Isolates #260, 263, 265, 268, 270, 272, and the unknown *Bacteroides* MAG bin2 formed a second clade, distinct from the *B. pyogenes* isolates (Fig. 2). These *Bacteroides* isolates form a clade in the phylogenetic tree that is close to *B. heparinolyticus* (GCF_004342845.1) and *Bacteroides zoogleoformans* (GCF_002998435.1) (Fig. 2, blue box). Whole genome ANI values between these *Bacteroides* isolates and *B. heparinolyticus* or *B. zoogleoformans* are 91% and 86%, respectively (Fig. S1B). ANI values below 95% to any characterized species of *Bacteroides* indicate that these isolates cannot be assigned to a known species of *Bacteroides*.

To independently confirm the results of this taxonomic analysis of both *Bacteroides* clusters, genome sequences were also analyzed using the TYGS (dsmz.de). The results of the TYGS whole genome analysis were identical to those obtained using GTDB-tk. Taken together, phylogenomic analyses indicate that the *Bacteroides* in these LA samples are *B. pyogenes* and a previously unknown species of *Bacteroides* that we propose be named Candidatus *Bacteroides purulensis* due to this novel species originating from a purulent infection.

### Analysis of metabolic potential in LA *Bacteroides*

**Fig 3:**
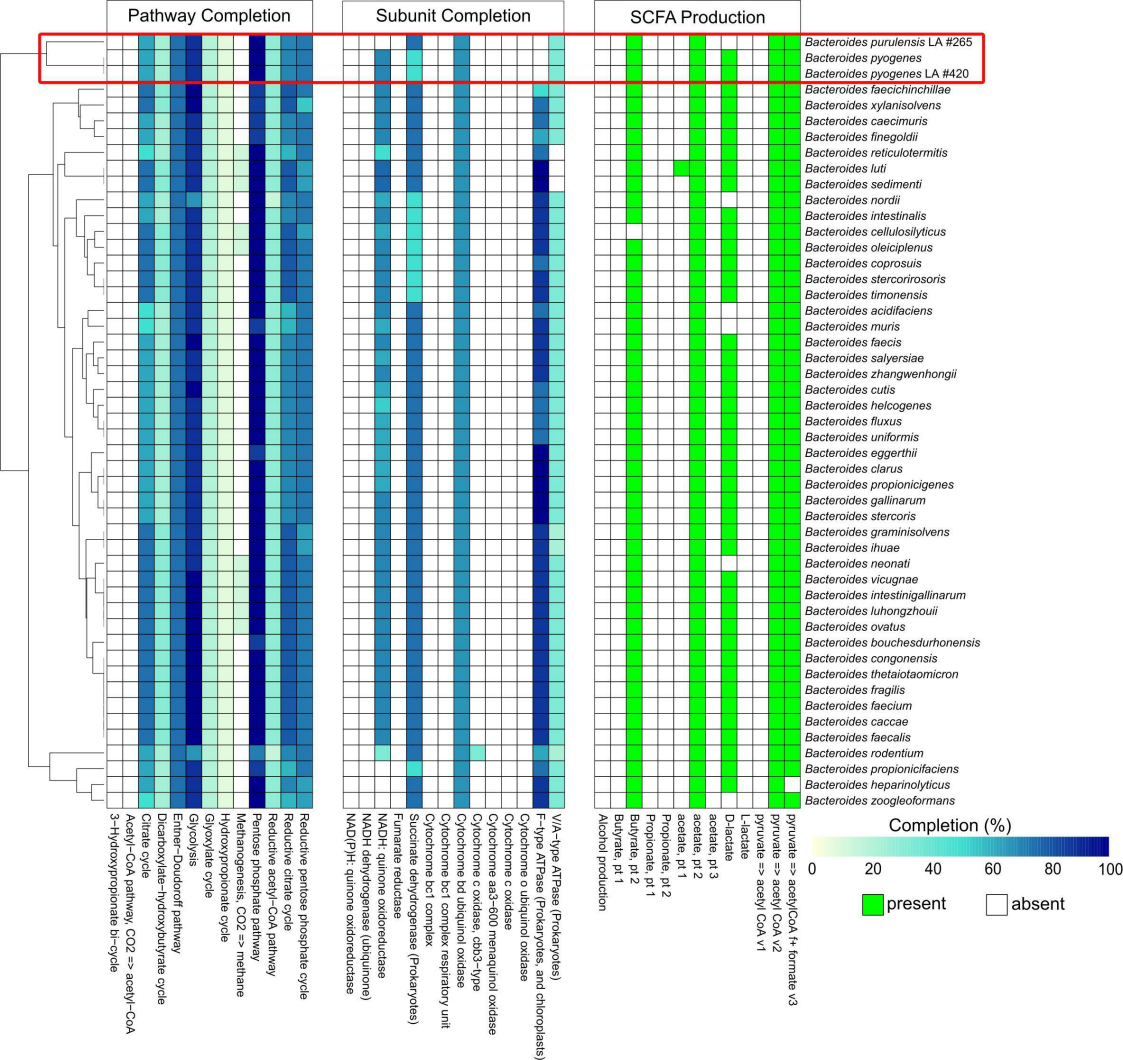
Comparison of metabolic pathways, enzyme subunit completion, and short-chain fatty acid (SCFA) production potential within *B. purulensis* and *B. pyogenes* isolates to type strains of *Bacteroides* species. Hierarchical clustering is based on the dissimilarity between pathway completeness, enzyme subunit completeness, and the presence/absence of metabolic pathways.

To simplify downstream genomic analysis of LA *Bacteroides*, a representative genome assembly was chosen for detailed examination of the metabolic and functional capacity of the isolates. The genome assemblies for *B. pyogenes* isolate #420 and *B. purulensis* isolate #265 were used for this analysis. A comparison of the metabolic pathways encoded in the genomes of these LA isolates to a range of *Bacteroide*s species found a high level of conservation in the metabolic pathways found in *Bacteroides*. Specifically, central metabolic pathways, including glycolysis, pentose phosphate, citrate cycles, and the Entner-Doudoroff pathway are highly conserved (Fig. 3). Intriguingly, F-type ATPase and NADH:quinone oxidoreductase, enzymes that are involved in oxidative phosphorylation and are found in other *Bacteroides* species, were absent in both *B. pyogenes* and *B. purulensis*. All of the species of *Bacteroide*s that were included in the analysis have complete pathways to produce the short-chain fatty acid acetate and convert pyruvate into acetyl-CoA (Fig. 3). Most species also have the pathways required to produce butyrate and formate. *B. purulensis* is one of the few species that does not appear to be capable of producing D-lactate. COG classification of the genomes of this same group of *Bacteroides* was also used to assess their functional capacity (Fig. S2). Interestingly, hierarchical clustering of genomes based on the relatedness of their COG contents shows that *B. pyogenes* and *B. purulensis* cluster together in the resulting dendrogram. Similarly, hierarchical clustering based on the DRAM analysis also resulted in the *Bacteroides* that were isolated from LA clustering together (Fig. 3). This analysis suggests that these bacteria may have similar functional roles in abscesses.

### Examining the diversity of CAZymes encoded by *Bacteroides*

**Fig 4:**
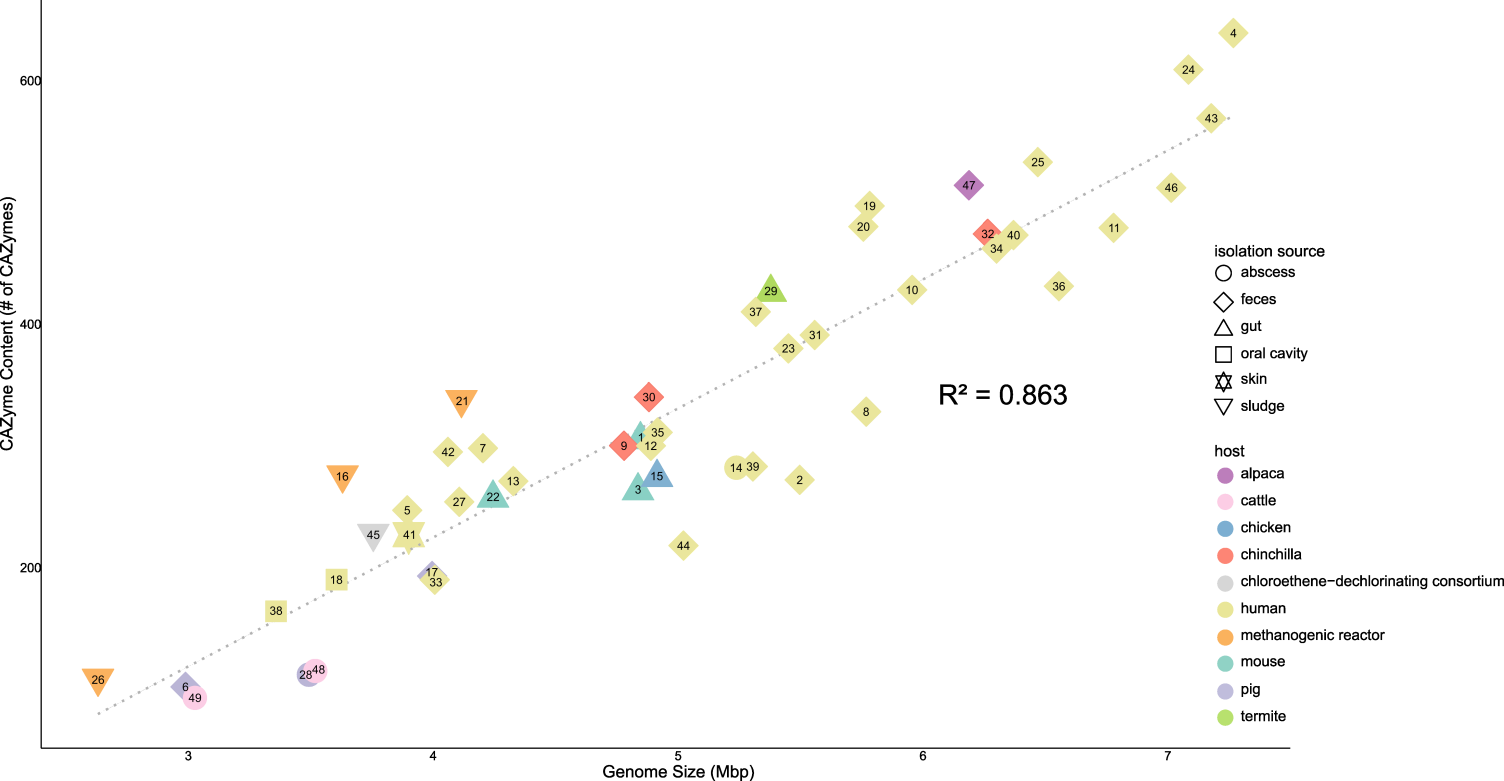
Relationship between genome size and the carbohydrate-active enzyme content within the genus *Bacteroides*. Each data point is numbered and is from one of the *Bacteroides* species included in the phylogenomic analysis (Data S3). The shape of the data points corresponds to the isolation source and is colored based on the host from which the isolate was obtained.

*Bacteroides* are well known for their ability to utilize carbohydrates, and characterizing the CAZymes encoded in their genome, collectively referred to as the CAZome, can provide clues about the *in vivo* metabolic roles of these bacteria. To contextualize carbohydrate metabolism of *Bacteroides* found in LAs, the CAZomes of all *Bacteroides* strains included in the DRAM analysis shown in Fig. 3 were evaluated. There is a strong positive correlation (*R*^2^ = 0.869) between genome size and CAZome content (Fig. 4). *Bacteroides* species with the largest CAZomes also had large genomes and were typically isolated from the gastrointestinal tract (*i.e*., gut and feces). The model gut bacterium *B. thetaiotaomicron* has a large genome (6.3 Mb) and encodes a large contingent of 463 CAZymes. In contrast, *Bacteroides* species that had smaller CAZomes also had small genomes and were isolated from non-gut environments, including abscesses and the oral cavity. This includes isolates from abscesses *B. pyogenes* (3.4 Mb, 113 CAZymes, type strain), *B. pyogenes* LA isolate #420 (3.5 Mb, 116 CAZymes, this study), and *B. purulensis* LA isolate #265 (3.0 Mb, 94 CAZymes), as well as the oral isolates: *B. heparinolyticus* (3.6 Mb, 191 CAZymes; type strain) and *B. zoogleoformans* (3.4 Mb, 165 CAZymes; type strain).

**Fig 5:**
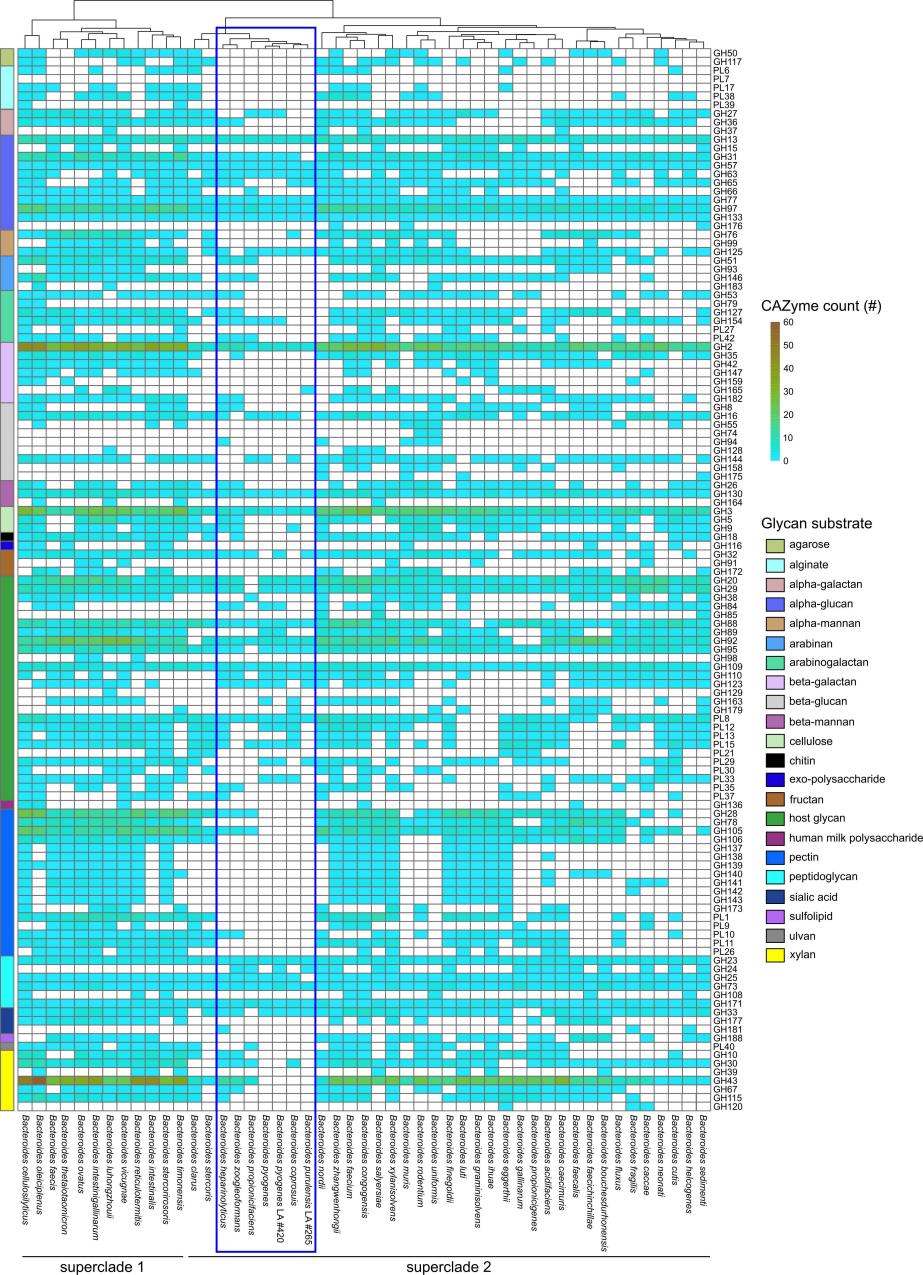
Carbohydrate degradation potential of *B. purulensis* and *B. pyogenes*, in relation to *Bacteroides* type strains. Enzymes belonging to the GH and PL families were identified using dbCAN3 and were grouped based on predicted activity according to dbCAN-sub (59) and the CAZy database (76). The number of enzymes within each CAZyme family is displayed as a heatmap, and the dissimilarity between CAZome content was used to cluster the *Bacteroides* strains.

To identify the potential ability to degrade diverse carbohydrate polymers by a range of *Bacteroides* species, the glycoside hydrolase (GH) and polysaccharide lyase (PL) CAZyme contents were assessed (Fig. 5). Hierarchical clustering based on the dissimilarity in the CAZomes identified two major *Bacteroides* superclades in the dendrogram. Superclade 1 (Fig. 5, left) was exclusive to gut isolates, while superclade 2 (Fig. 5, right) included species from both gut and non-gut environments. A core set of GHs was present in all *Bacteroides* species, and these included GH2, GH3, GH13, GH23, GH57, GH73, GH77, GH97, GH109, GH130, GH133, and GH171. Gut isolates within superclade 1 have enzymes that would make them highly adept in the degradation of most carbohydrates, including plant cell wall and seaweed polysaccharides (i.e., agarose, alginate, and ulvan). The liver abscess isolates grouped together within superclade 2 (Fig. 5, red box) and clustered with non-gut *Bacteroides: B. coprosuis, B. pyogenes, B. propionicifaciens, B. heparinolyticus, and B. zoogleoformans*. The liver abscess isolates primarily possessed CAZymes that are required for the degradation of α-glucans (e.g., starch, glycogen, and pullulan), host glycans (e.g., GAGs and mucin), and bacterial cell wall peptidoglycan (Fig. 5).

**Fig 6:**
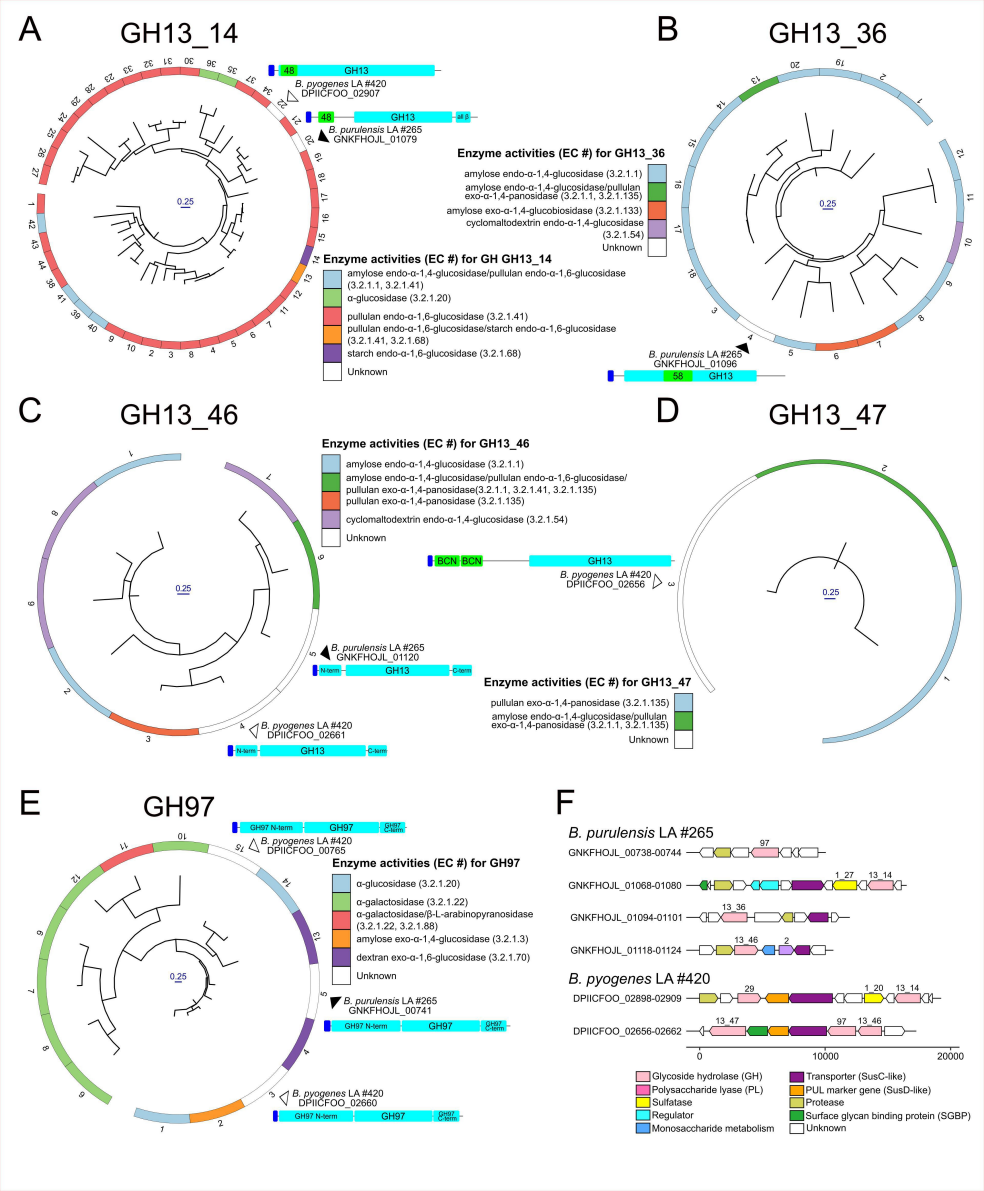
Phylogenetic trees of CAZy families (**A**) GH13_14, (**B**) GH13_36, (**C**) GH13_46, (**D**) GH13_47, and (**E**) GH97 that are predicted to be extracellular starch and/or glycogen-degrading enzymes in *B. purulensis* LA isolate #265 (black triangle) and *B. pyogenes* LA isolate #420 (white triangle). Genome schematics are to scale and color coded for signal peptide (dark blue), glycoside hydrolase (GH) (light blue), and carbohydrate-binding module (CBM) or CBM-like (green). CAZy database annotated enzyme function and corresponding EC number are represented in the outer ring and are color coded based on the legend for each CAZyme phylogenetic tree. (**F**) Gene clusters and PULs containing enzymes that putatively target glycogen and/or starch.

Enzymes targeting starch/glycogen and host-derived glycans could play a role in colonizing and surviving in the liver. Therefore, we focused our CAZyme analysis on these enzyme activities. Most CAZy families display polyspecific enzyme activity, so to more accurately predict the function of CAZymes encoded by *B. purulensis* and *B. pyogenes* that may target glycogen/starch and host-derived glycans, phylogenetic analysis of CAZy families was carried out. Both species of *Bacteroides* isolated from LA encoded intracellular GH13_8, GH13_38, GH57, GH77, and GH133 enzymes, as well as secreted GH13_14, GH13_46, and GH97 enzymes (Data S2 and S4, respectively). Additionally, *B. purulensis* encodes a secreted GH13_36, and *B. pyogenes* encodes a secreted GH13_47. Based on high levels of identity to characterized enzymes found in *B. thetaiotaomicron,* it can be inferred that these GH13s possess a range of activities involved in starch and/or glycogen deconstruction, including pullulan endo-α-1,6-glucosidase, amylose endo-α-1,4-glucosidase, cyclomaltodextrin endo-α-1,4-glucosidase, and pullulan exo-α-1,4-panosidase activities (Fig. 6). The GH97s are predicted to have α-glucosidase and amylose exo-α-1,4-glucosidase activity.

**Fig 7:**
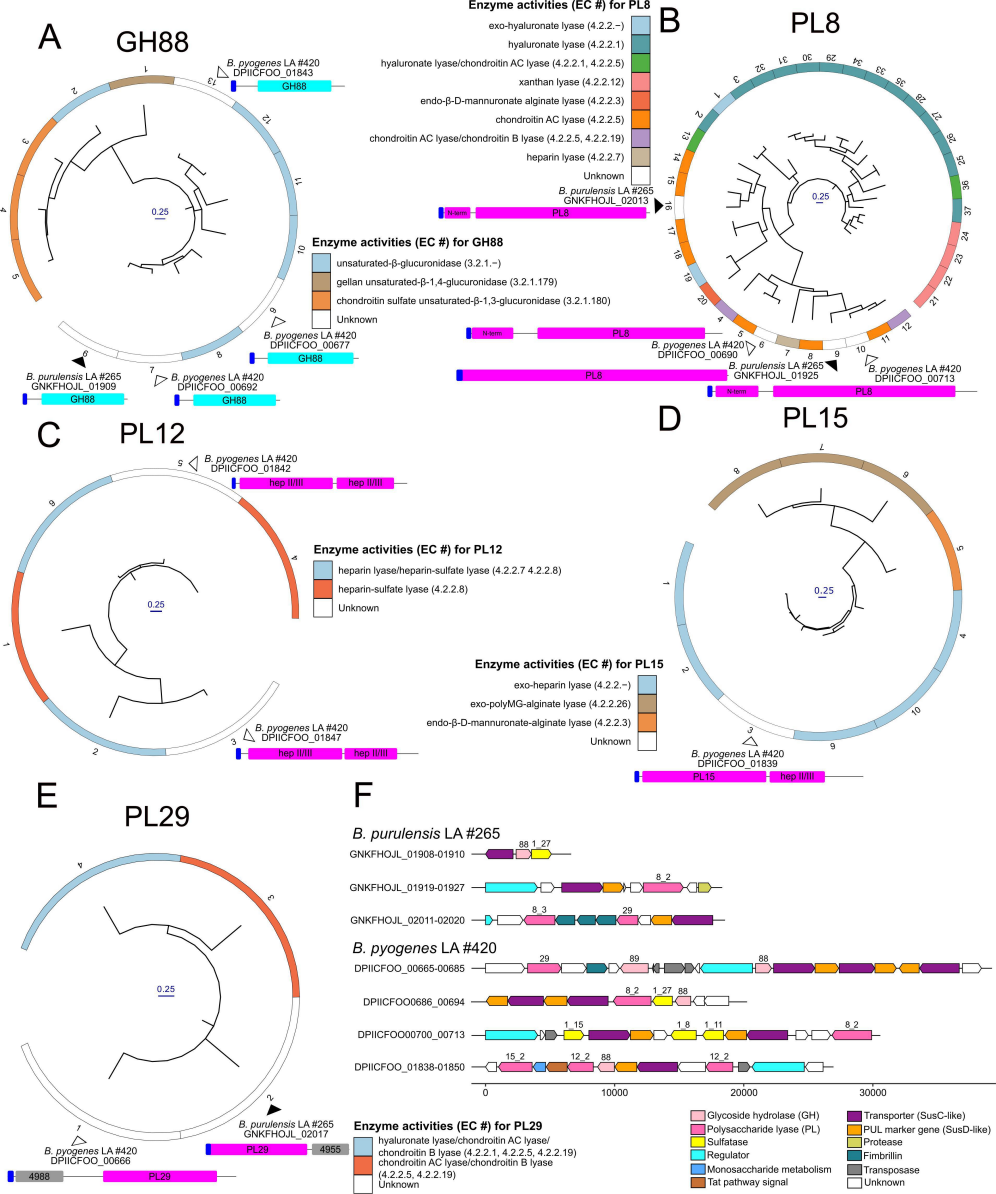
Phylogenetic trees of CAZy family (**A**) GH88, (**B**) PL8, (**C**) PL12, (**D**) PL15, and (**E**) PL29 that are predicted to be extracellular glycosaminoglycan-degrading enzymes in *B. purulensis* LA isolate #265 (black triangle) and *B. pyogenes* LA isolate #420 (white triangle). Gene schematics are to scale and color-coded for signal peptide (dark blue), glycoside hydrolase (GH) (light blue), polysaccharide lyase (purple), and unknown domains (gray). CAZy database annotated enzyme function and corresponding EC number are represented in the outer ring and are color-coded based on the legend for each CAZyme phylogenetic tree. (**F**) Gene clusters and PULs containing enzymes that putatively target glycosaminoglycans.

Both species of *Bacteroides* LA isolates also encode a range of CAZymes targeting host-derived GAGs that may be present in the liver, including heparan sulfate, heparin, chondroitin sulfate, and hyaluronic acid. Interestingly, the presence/absence of CAZymes with known activity against GAGs predicts that the substrate specificity of each species differs. *B. purulensis* lacks heparin lyases (PL12 and PL15), whereas *B. pyogenes* contains all the PLs, GHs, and sulfatases required to effectively degrade heparin/heparan sulfate (Fig. 7C, D, and F). Instead, *B. purulensis* encodes lyases that are predicted to act on chondroitin sulfate (PL8 and PL29) (Fig. 7B, E, and F). In addition to differences in the predicted CAZyme content of each LA isolate, host glycan-targeting enzymes localize to unique sites in the phylogenetic trees, also suggestive of distinct substrate specificities in the enzymes encoded by each *Bacteroides* species. As is commonly observed in *Bacteroides*, most carbohydrate-metabolizing genes are organized into polysaccharide utilization loci in *B. purulensis* and *B. pyogenes* (Fig. 6F and 7F); however, the gene composition of these operons was not conserved, and they are more frequently observed in *B. pyogenes*. The presence of SusC/D pairs predicted 28 PULs in the genome of *B. purulensis*, compared to 42 PULs in *B. pyogenes* (Data S2). The differences in substrate specificity predicted for the CAZymes and unique PUL structures (Fig. 6F) both provide evidence of unique carbohydrate-metabolizing capacity in *B. purulensis* and *B. pyogenes* LA isolates.

**Fig 8:**
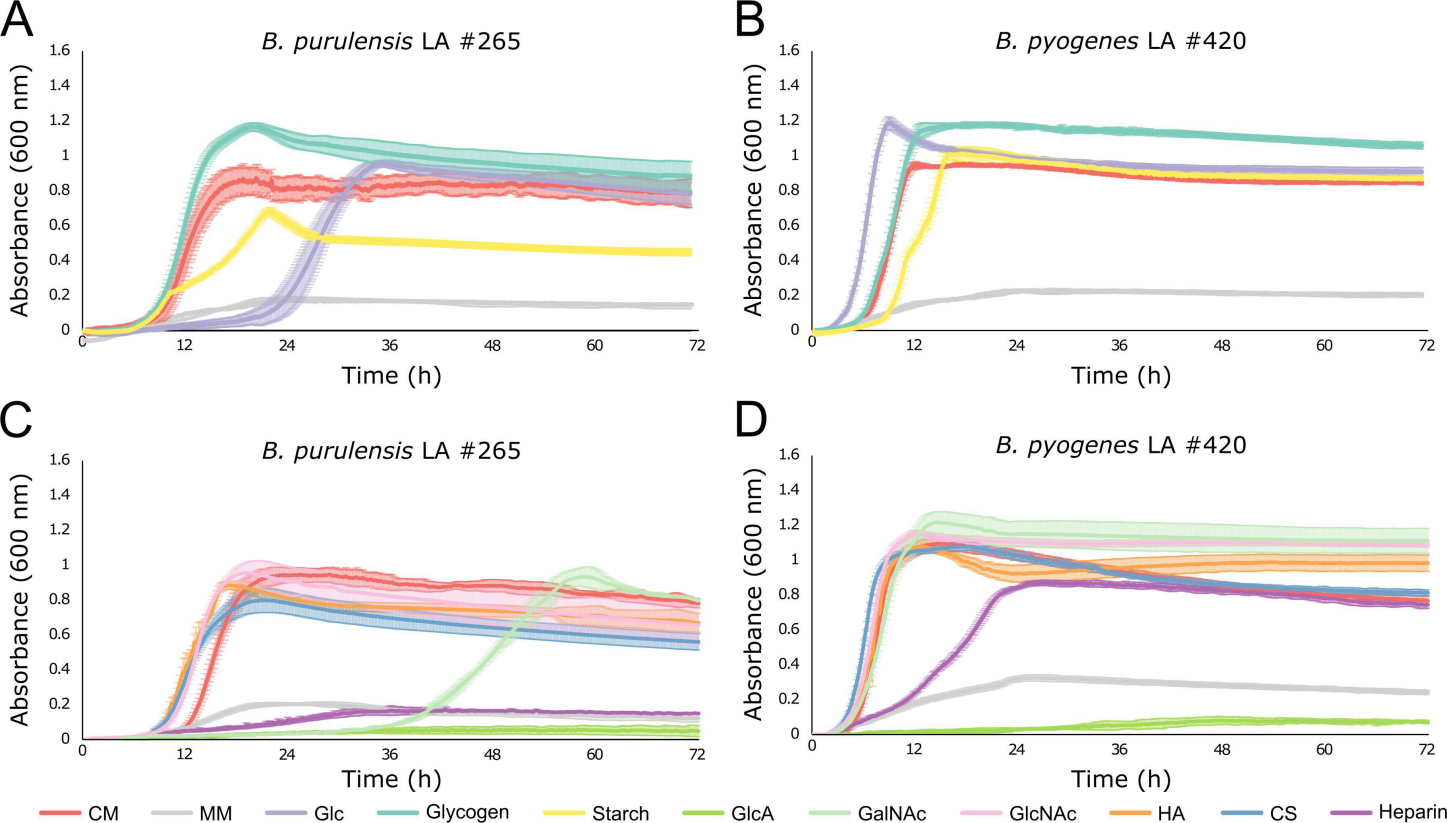
Growth profiles of *Bacteroides purulensis* LA isolate #265 (A and C) and *Bacteroides pyogenes* LA isolate #420 (B and D) in a minimal media supplemented with the α-glucan substrates (0.5% [wt/vol] final concentration): glucose (Glc), glycogen, or soluble starch (A and B), or the glycosaminoglycan substrates: GlcA, GalNAc, GlcNAc, HA, CS, or heparin (C and D), in comparison to growth in MM without glycan supplementation and in the rich medium, customized chopped meat (*n* = 4).

*B. purulensis* and *B. pyogenes* were cultured to evaluate their ability to catabolize different types of α-glucan and GAG substrates. For α-glucan substrates, glucose (Glc), glycogen, and soluble starch were investigated. Both species could rapidly utilize glucose and glycogen but grew less efficiently on starch (Fig. 8A and B). Interestingly, *B. purulensis* showed preference for glycogen in comparison to starch and glucose. *B. purulensis* grew to approximately twice the cell density of the starch growth conditions in the presence of glycogen (Fig. 8A). While the final cell density of *B. purulensis* in the glucose condition was similar to that of glycogen, there was a significantly longer lag phase with glucose (28 hours). Both species displayed a biphasic growth phenotype when starch was provided as the carbon source but not when glycogen was used. For GAG substrates, glucuronic acid (GlcA), N-acetylgalactosamine (GalNAc), N-acetylglucosamine (GlcNAc), HA, CS, and heparin were tested. Both species utilized the amino monosaccharide components (GalNAc and GlcNAC) found in GAGs but not the uronic acid component, GlcA (Fig. 8C and D). Both species rapidly metabolized the GAG polysaccharides HA and CS; however, only *B. pyogenes* was able to utilize heparin (Fig. 8D). Although *B. pyogenes* could grow on heparin sulfate, it had a significantly longer lag phase compared to HA and CS. These phenotypes could be replicated in all isolates.

### Presence of virulence factors and antimicrobial resistance genes

The presence of potential VFs encoded by both LA *B. purulensis* and *B. pyogenes* was investigated. A blastp search against the virulence factor database identified 20 putative VFs that were conserved in both *Bacteroides* species. Most of the VFs were associated with immune modulation, stress survival, and bacterial adherence (Data S5). An additional four VFs involved in biofilm formation and adhesion were found to be unique to *B. pyogenes*. There were eight VFs that were unique to *B. purulensis,* including proteins involved in capsular polysaccharide synthesis, transcriptional regulation, immune evasion, cytolysins, and transcriptional regulation or expression of cytolysins. Several proteins that may also play a role in bacterial cell adhesion were identified and annotated as fimbrillin-like proteins and adhesins. GH33 sialidases were identified in both species and represent an additional mechanism with which these pathogens can modify host glycans. Protease genes were abundant in both bacteria, with 47 metallopeptidases, 36 serine peptidases, and 10 cysteine peptidases encoded in the genome of *B. purulensis*, and 54 metallopeptidases, 32 serine peptidases, and 14 cysteine peptidases encoded in the genome of *B. pyogenes* (Data S2). Of note, several M60 peptidases commonly associated with mucin degradation and two collagenases within the U32 family of peptidases were found in both species.

The presence of antimicrobial resistance (AMR) genes in both LA *Bacteroides* species was also investigated. *B. purulensis* is predicted to be resistant to tetracycline, based on the presence of the Tet(Q) resistance ribosomal protection protein (Data S2). In contrast, no AMR genes listed in the NCBI Bacterial AMR Reference Gene Database were identified in *B. pyogenes*. Both *Bacteroides* genomes encoded five proteins identified as multidrug and toxic compound extrusion efflux pumps, and several other efflux pumps that could potentially target antimicrobials (Data S2). The enrichment medium that was used contained vancomycin, and both species could be isolated from enrichment culture and direct plating on non-selective media. This indicates that both species have some resistance to glycopeptide antibiotics.

## DISCUSSION

The welfare of cattle and the economic sustainability of beef production are both significantly impacted by the rising prevalence of liver abscesses. The prevailing hypothesis on how LAs form suggests that acidotic conditions from rapid starch fermentation disrupt the integrity of the rumen and/or hindgut epithelium. This epithelial damage enables the translocation of gut microbes to the liver via the portal vein, where they colonize and form an abscess (6). Advances in next-generation sequencing have substantially improved our understanding of the poly-microbial nature of LA in cattle. Although *F. necrophorum* is considered the primary infectious agent, recent evidence points to a polymicrobial community in LAs (6–10). Studies have reported two distinct LA community subtypes in severely abscessed livers: one dominated by *Fusobacterium* (*Fusobacteriota*) and the other by *Bacteroidetes* (*Bacteroidota*)-dominated subtype (6, 9, 10). Our laboratory recently identified *Bacteroides* as the second most abundant organism in >55% of the LAs collected from cattle with and without the administration of in-feed tylosin (7). Because the short reads generated in 16S rRNA amplicon sequencing cannot reliably identify sequence variants beyond the genus level, the specific *Bacteroides* species that are present in LAs and their functional roles in pathogenesis are unknown. Developing novel technologies to reduce the incidence of LAs requires a detailed understanding of abscess microbiology and the role each bacterial species plays in their formation. To address this knowledge gap, we have isolated, identified, and sequenced the genomes of two species of *Bacteroides* from the purulent material in severe liver abscesses, *B. pyogenes* and a previously unknown species, Candidatus *Bacteroides purulensis*.

No additional *Bacteroides* species were isolated or identified in our metagenomic sequencing data. It is possible that more species may be involved in the development of LAs, and these were not recovered. Oxygen exposure during abscess dissection and sample processing, inter-animal variability, and a small sample size may have limited the detection of other species (6, 7). Notably, we did not identify *B. fragilis* in these abscess samples; however, it is commonly identified in a variety of clinical abscess samples, including intra-abdominal and liver abscesses in humans (17, 20). Future efforts should focus on isolating *Bacteroides* from a larger number of abscesses and minimizing the time that samples are exposed to oxygen.

The co-occurrence of *Bacteroides* and *Fusobacterium* has been observed in a variety of infections. Metritis is a uterine inflammatory disease caused by a polymicrobial infection involving *Bacteroides* (i.e*., B. heparinolyticus* and *B. pyogenes*) (77)*, Porphyromonas*, and *Fusobacterium* species (i.e*., F. necrophorum*). Interestingly, the co-occurrence of *B. heparinolyticus* and *F. necrophorum* was positively correlated with metritis severity in dairy cattle (78). *Bacteroides* and *Fusobacterium* bacteria strongly interacted with each other in the blood but not in the feces and vaginal environments where they were also present, and it was speculated that blood could be a gateway for these metritis-causing pathogens to enter the uterine environment during calving (78). Further studies identified 152 metabolites that differed between cows that developed postpartum metritis and those that did not (79). Metabolites from lysine metabolism and microbial fermentation of aromatic amino acids were associated with inflammation and *Bacteroides* (79). Inflammation in the oral cavity of cattle with periodontitis is also associated with *Fusobacterium*, *Prevotella,* and *Bacteroides* (80). Interestingly, the adherence of *Bacteroides* to gingival epithelial cells is associated with periodontal tissue destruction (81). The observation that *Bacteroides* and *F. necrophorum* co-occur in a range of infections indicates that there may be a synergistic relationship between these organisms that contributes to abscess development.

Members of *Bacteroides* are well known for their ability to metabolize carbohydrates (82) and can adjust their metabolism in response to changes in nutrient availability within an ecosystem (83, 84). Examining the polysaccharide-degrading capacity of the *Bacteroides* isolated from LA may provide insight into whether these bacteria metabolize glycans in the liver. This analysis found that *B. purulensis* and *B. pyogenes* were more similar to *Bacteroides* isolated from the oral cavity and abscesses than to those isolated from the gastrointestinal tract. They possess significantly smaller genomes and CAZomes, as compared to *Bacteroides* inhabiting the gut, such as *B. thetaiotaomicron*. Approximately 4.6% of the *B. thetaiotaomicron* genome consists of CAZymes, enabling this microbe to utilize a broad range of complex carbohydrates (85) (Fig. 5). In comparison, 1.8% and 1.9% of the *B. purulensis* and *B. pyogenes* genomes, respectively, encode CAZymes that are predicted to primarily target α-glucans, host glycans, and peptidoglycan (86). Glycogen is one of the most abundant α-glucans found in the liver (87), while GAGs and proteoglycans are abundant in the extracellular matrix of liver tissue (88). Based on the genomic data and our *in vivo* carbohydrate utilization assays, the *Bacteroides* present in LAs are likely metabolizing glycogen and GAGs present within the liver (89). We hypothesize that this may contribute to bacterial survival and tissue invasion during the development of liver abscesses.

Glycogen accounts for ~1% of the tissue wet weight of liver tissue in cattle (87) and serves as a flexible, transient nutrient source that can be degraded quickly to maintain blood glucose levels (90). Glycogen is also hypothesized to serve as an energy source for bacteria that invade the liver (91), but the mechanism by which bacteria utilize host-derived glycogen remains unclear. A previous genomic analysis of 55 bacterial species revealed that those lacking glycogen-metabolizing enzymes (i.e*.,* GT5, GT35, and GH13) generally displayed parasitic or symbiotic behavior (91). Intriguingly, glycogen catabolic and anabolic enzymes were both found in the genomes of *B. purulensis* and *B. pyogenes*. In addition to catabolizing glycogen as an energy source, it may be beneficial for these isolates to synthesize and store glycogen intracellularly (92). This trait has been linked to colonization persistence (93, 94) as it provides an energy reserve in unfavorable or changing environments (95). Utilization of host-derived glycogen has been observed in both commensal and pathogenic bacteria. Pullulanases (GH13s) from human-vaginal isolates and from *Streptococcus pneumoniae* have been characterized and identified as responsible for glycogen and pullulan degradation (96, 97). *B. purulensis* and *B. pyogenes* both contain secreted GH13_14 pullulanases that are predicted to be active on the α-1,6 glycosidic linkages present in glycogen (Fig. 6A). The presence of other α-glucan-targeting CAZymes (e.g., other GH13s and GH97s) in both species would enable complete degradation of glycogen and maltooligosaccharides (98, 99). Interestingly, while both *B. purulensis* and *B. pyogenes* grew well when glycogen or glucose was provided as a carbon source, both organisms were less efficient at metabolizing starch. Compared to starch, glycogen is more branched, has higher solubility, and is less crystalline. The ability of both of these organisms to metabolize glycogen, the primary carbohydrate storage molecule found in the liver, supports the hypothesis that they are utilizing host-derived glycogen *in vivo*. While further studies are required to characterize the mechanism behind the specificity for glycogen over starch, it may be a result of the differences in the physiochemical properties of these carbohydrate storage molecules.

Heparin and other GAGs, such as heparan sulfate and chondroitin sulfate, are host-derived glycans that are also plausible energy sources for *B. pyogenes* and *B. purulensis* within the liver. HS and CS proteoglycans are present in the ECM, and shedding proteoglycans from the surface of hepatocyte cells by heparanases has been found to contribute to liver disease pathology (100–103). In contrast to the GAGs present in the ECM, heparin is exclusively synthesized by connective-tissue-type mast cells (MCs) and stored within granules along with CS, proteases (e.g., tryptase and chymase), and cytokines (104, 105). Degranulation/activation of MCs has been associated with liver disease in humans (i.e., hepatitis, cirrhosis, and hepatocellular carcinoma) (106), skin abscesses caused by *Staphylococcus aureus* (107), and metritis in dairy cattle (108). Whether there is a potential role that mast cells and GAGs play in the development of liver abscesses remains unknown.

GAGs play an important role in the regulation of numerous physiological processes, such as cell signaling and adhesion, wound healing, and host-pathogen interactions (109–112). The closely related bacterium, *B. heparinolyticus*, produces a potent heparinase, which can digest host GAGs that play essential roles in cell-cell adhesion and tissue integrity (113). PULs targeting heparin/HS (hep/HS) CS and dermatan sulfate (DS) GAGs have been described in numerous *Bacteroides* species, and those targeting HS have a similar composition to a predicted heparin PUL (Fig. 7E) that was observed in *B. pyogenes* (89, 114). In agreement with the genomic analysis, *B. pyogenes* grew effectively on all three of these GAGs. Interestingly, the growth of *B. pyogenes* was impaired when grown on highly sulfated HS but not with CS/DS, GAGs with low levels of sulfation. This result suggests that GAG sulfation level impacts the rate of metabolism by *B. pyogenes*. Intriguingly, no sulfatases are present in the *B. pyogenes* hep/HS PUL, which suggests that these bacteria may target low-sulfated regions of HS or encode novel sulfatase enzymes. In contrast, *B. purulensis* lacks several of the enzymes required to deconstruct HS, and it was unable to grow when HS was provided as a carbon source. The presence of PL8s that have been described within CS and DS PULs in the genome of *B*. *purulensis* enabled it to effectively utilize CS and DS (Fig. 7E) (89). We hypothesize that these *Bacteroides* utilize GAGs in the ECM of liver tissue as a nutrient source and that this may contribute to the development of LAs by disrupting cell-cell adhesion and tissue integrity. The differences in GAG specificity observed between *B. pyogenes* and *B. purulensis* would enable them to target different nutrient sources *in vivo*, thereby reducing direct competition.

Bacterial pathogens encode a range of virulence factors that play essential roles in their virulence. *B. fragilis* is the most well-characterized opportunistic pathogen within the genus *Bacteroides,* and it possesses an extensive range of VFs, including multidrug resistance, a zinc-dependent metalloprotease (fragilysin), hemolysin, cytolysin, neuraminidase, and extracellular hydrolytic enzymes that can break down the ECM (17). The presence of adhesins and capsular polysaccharides also contributes to virulence by facilitating bacterial colonization and conferring resistance to oxidative stress (17, 21). Unfortunately, species of *Bacteroides* that are known to cause infections, such as *B. fragilis*, are not represented in the VFDB, and this lack of representation complicated the identification of VFs in *B. purulensis* and *B. pyogenes*. Despite these challenges, we putatively identified 21 VFs that are conserved between the species and seven VFs that are unique to either *B. purulensis* or *B. pyogenes*. Additionally, both species encoded over 90 genes encoding proteases, including metalloproteases, collagenase, serine peptidases, and cysteine peptidases. These proteases may function both during the degradation of the ECM and the destruction of liver tissue (115). *B. purulensis* encodes two genes for thiol-activated cytolysins, which may contribute to virulence by lysing host immune cells (116). Leukotoxin and cytolysins are known to be essential VFs in *F. necrophorum* (117), and the presence of similar proteins in *B. purulensis* underscores the important role these proteins serve during the establishment of intra-abdominal abscesses. Several other VFs associated with cell adhesion, stress survival, and immune modulation were also found in both LA *Bacteroides*. Antimicrobial resistance is another important VF frequently observed in clinical isolates of *B. fragilis*, which are typically resistant to several antimicrobials (118). While both organisms were resistant to vancomycin, *B. purulensis* was predicted to be resistant to tetracycline, while *B. pyogenes* did not encode known antimicrobial resistance genes. The presence of several efflux pump proteins may confer some resistance, as similar mechanisms have been described in *B. fragilis* (119). Antimicrobial susceptibility studies are required to conclusively assess the AMR profile of these microbes.

### Conclusion

We have identified, isolated, and genomically characterized two species of *Bacteroides* from bovine liver abscesses. These data provide the first conclusive identification of these organisms, including the discovery of a previously unknown species, Candidatus *Bacteroides purulensis*. While additional studies are required to fully understand the virulence mechanisms and bacterial interactions that contribute to abscess formation, these data provide a critical foundation for expanding our knowledge of the potential role *Bacteroides* plays in this process and could contribute to the identification of novel targets for developing treatments to prevent this important production-limiting disease.

## Data Availability

Sequence data and genome assemblies were submitted to NCBI under BioProject PRJNA1114737. Metagenomic sequence data were submitted under BioProject PRJNA1110759.

## SUPPLEMENTAL MATERIAL

Data S1 spectrum.00423-25-s0001.xlsx Differentially abundant functions.

Data S2 spectrum.00423-25-s0002.xlsx Genome annotations.

Data S3 spectrum.00423-25-s0003.xlsx Relationship between genome size and CAZyme content in *Bacteroides*.

Data S4 spectrum.00423-25-s0004.xlsx Enzyme activity & localization prediction.

Data S5 spectrum.00423-25-s0005.xlsx Predicted virulence factors.

Supplemental figures spectrum.00423-25-s0006.docx Figures S1 and S2.

ASM does not own the copyrights to Supplemental Material that may be linked to, or accessed through, an article. The authors have granted ASM a non-exclusive, world-wide license to publish the Supplemental Material files. Please contact the corresponding author directly for reuse.
