# Supplementary material for: Unraveling the microbial diversity of bovine liver abscesses: isolation, identification, and genomic characterization of the Bacteroides found in hepatic lesions
Source: Microbiol Spectr. 2025 Apr 17;13(6):e00423-25. doi: 10.1128/spectrum.00423-25 (PMC12131861; doi:10.1128/spectrum.00423-25)
Supplement: Supplemental figures — Figures S1 and S2. [file spectrum.00423-25-s0006.docx]

**Supplementary Figures**


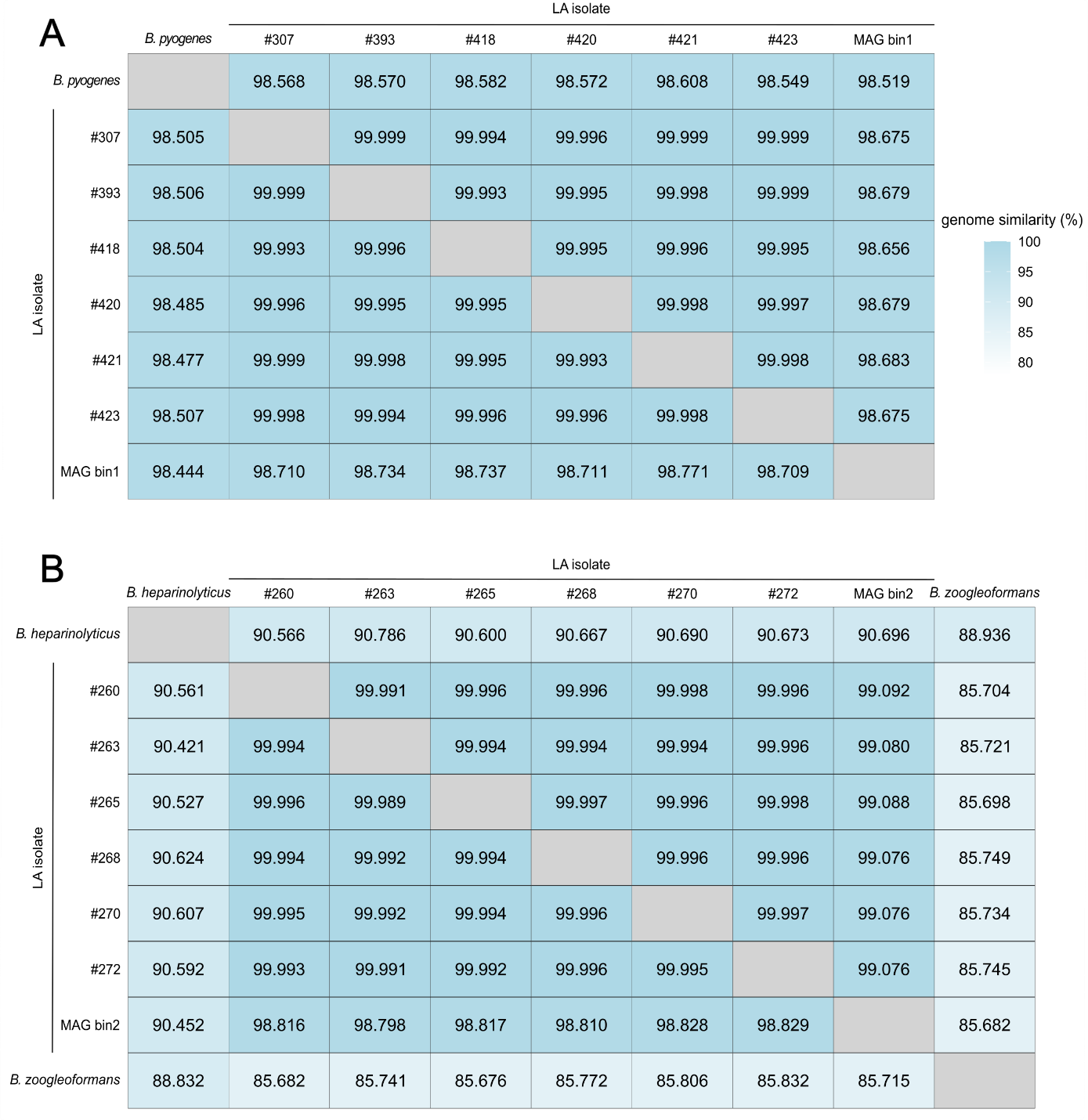


**Supplementary Figure 1:** Average nucleotide identity (ANI) matrix for of *Bacteroides* isolates and MAGS collected from abscessed liver tissue of cattle, in relation to the closest phylogenetic *Bacteroides* type strains. (A) Where the LA isolates (#307, 393, 418, 420, 421, 423) and MAG bin1 determined to be *B. pyogenes* and the *B. pyogenes* type strain, and (B) the unknown *Bacteroides* isolates (#260, 263, 265, 268, 270, 272) and MAG bin2 with the *B. zoogleoformans* and *B. heparinolyticus* type strains. An ANI threshold of ≥ 95% similarity was used for demarcating species (Jain, Rodriguez-R et al. 2018).


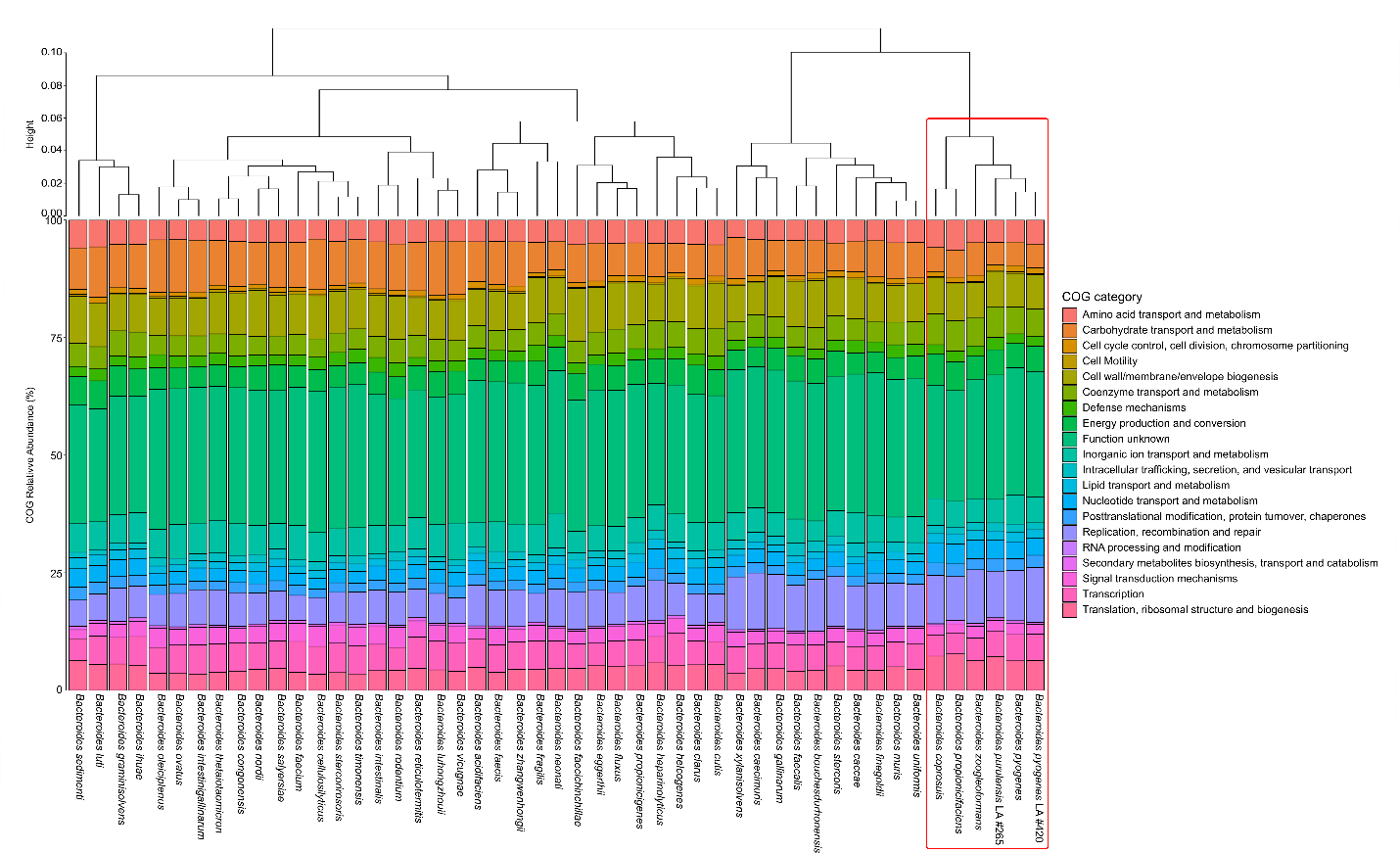


**Supplementary Figure 2:** Relative abundance of Clusters of Orthologous Groups of proteins (COGs) present within *Bacteroides purulensis* and *Bacteroides pyogenes* isolated from LA (red box)*,* and *Bacteroides* type strains. Hierarchical clustering of *Bacteroides* species is based on the relatedness of their COG contents.
